# Translation and psychometric analysis of urdu version of modified polycystic ovary syndrome health related quality of life questionnaire (MPCOSQ-U)

**DOI:** 10.1186/s12905-024-03266-x

**Published:** 2024-07-23

**Authors:** Rabbiya Zaman, Sarah Ehsan, Abeer Fatima, Sumaiyah Obaid, Javeria Shahzadi

**Affiliations:** 1 Department of Physical Therapy, Zohra Institute of Health Sciences, Rawalpindi, Pakistan; 2 Faculty of Rehabilitation and Allied Health Sciences, Riphah International University, Islamabad, Pakistan

**Keywords:** Females, Gynecology, Polycystic ovary syndrome, Psychometrics, Quality of life

## Abstract

**Background:**

Polycystic ovary syndrome is a metabolic disorder prevalent among females of reproductive age. The symptoms of PCOS profoundly affect the quality of life of these females. Outcome measures specific to PCOS are crucial to the management of these patients. The MCPOSQ is a validated tool to measure the health-related quality of life specific to PCOS. The purpose of this study was to translate the modified polycystic ovary syndrome quality of life questionnaire (MPCOSQ) and to determine the reliability and validity of the modified polycystic ovary syndrome quality of life questionnaire Urdu version (MPCOSQ-U).

**Methodology:**

This cross-sectional study was conducted in Islamabad/Rawalpindi. The MPCOSQ was translated to Urdu and validated by expert gynaecologists. The MPCOSQ-U and SF-36 were administered to one hundred eighty females with PCOS. The MPCOSQ-U was evaluated for internal consistency, test-retest reliability, factor analysis, face validity, content validity and construct validity.

**Results:**

The average age (years) of the females was 25.27(1.83). The MPCOSQ-U showed excellent test-retest reliability and internal consistency (ICC_2,1_=0.95, Cronbach’s α = 0.97). The content validity index (CVI) was 0.92. There was a statistically significant but weak positive correlation between MPCOSQ-U and SF-36 (*r* = .186, *p* = .012).

**Conclusion:**

The Urdu version of the modified version of the polycystic ovarian syndrome quality of life questionnaire is a validated and reliable tool to assess the quality of life of Pakistani females with PCOS. This is an important step to cover the language barrier, which influences the outcome assessment in PCOS.

## Introduction

Polycystic ovary syndrome (PCOS) is a chronic, heterogeneous, metabolic disorder affecting females of reproductive age. PCOS Women manifest infertility, reduced insulin sensitivity, obesity, hirsutism, acne, and an increased risk of cardiovascular disorders and diabetes type 2 [1, 2]. Polycystic ovary syndrome can have different phenotype presentations based on clinical symptoms, biochemical tests and ultrasound. It has a strong impact on the overall life of females, as it is associated with numerous other comorbidities. [3, 4]

About 5–10% of the females in their reproductive age suffer from PCOS [5]. Although the prevalence varies according to the diagnostic criteria, PCOS is the most reported gynaecological disorder among premenopausal females, with a rising prevalence in Pakistan i.e. 55.41% in Karachi, 34.3% in Gujrat, and 48% in Hyderabad. [6–9]

Quality of life is an important outcome in PCOS because of its heterogenous nature. It affects both physical and mental health of the females. Numerous studies have reported a poor quality of life of females having PCOS. [10–13]

Previous research has used various outcome measures including Short form (SF-36), polycystic ovary syndrome questionnaire (PCOSQ-50), modified polycystic ovary syndrome quality of life questionnaire (MPCOSQ), Symptom checklist (SCL-90-R), and world health organization quality of life brief scale (WHOQOL-BREF) to assess the quality of life of females with PCOS. As concluded by a systematic review conducted by Moghadam et al., the use of a specialized instrument for PCOS has been shown to be more effective as it encompasses all disease-specific facets [14]. 

The influence of polycystic ovary syndrome on the quality of life of females necessitates the need for health-related quality of life (HRQOL) tool specific for the condition. Therefore, in 1998, Cronin et al. first developed the PCOSQ, which consists of 26 items related to PCOS-specific quality of life. These items were grouped into five subscales, i.e., emotions, body hair, weight, infertility, and menstrual problems. All items are scored on a 7-point Likert scale. There are several health-related questionnaires, but the PCOSQ is most accurate in measuring quality of life and perspectives among females and is said to be more adequate to rule out undiagnosed issues related to health. [15]

The PCOSQ was modified in 2007, as the subscale domains were found to be inadequate. Barnard et al. reintroduced the PCOSQ as the MPCOSQ, which consists of 30 items with 7 subscales. An additional acne subscale was added to the other six subscales. Higher scores indicate better quality of life. The MPCOSQ has been translated and validated into many languages, including German, Arabic, Austrian, Iranian, and Chinese. [6, 16–18]. In Pakistan the original version of PCOSQ has been translated into Pushto Language. [19]

Since quality of life is an important outcome in PCOS, there is a need for culturally sensitive patient reported outcome measure for PCOS females in Pakistan. Urdu is the national language of Pakistan; this study will enhance accessibility of MPCOSQ for Urdu-speaking individuals by overcoming the language barrier of the patients and improving their understanding of the questions. This will facilitate better research participation and will also be of clinical significance, providing a psychometrically sound tool for assessing the quality of life of Urdu-speaking females with PCOS.

The objective of this study was to translate the modified polycystic ovary syndrome quality of life questionnaire (MPCOSQ) and to determine the reliability and validity of the modified polycystic ovary syndrome quality of life questionnaire Urdu version (MPCOSQ-U).

## Methods

### Study design and participants

This cross-sectional study was conducted from November 2020 to August 2021 after approval from the Ethical Research Committee of Riphah International University (RIPHAH/RCRS/REC/00843). The Modified version of PCOSQ by Barnard et al. [16] was used in the study after their consent which was taken via email. The sample was recruited from the Zohra Institute of Health and Sciences and Islamabad Healing Centre using convenience sampling technique. We used an item to subject ratio for sample size estimation. Since there were 30 items in the MPCOSQ, using the subject to item ratio of 6:1 the estimated sample size was 180. This is a frequently employed method for sample size calculation in validation studies [20]. Females aged 18 to 30 years with a diagnosis of PCOS made on the Rotterdam Criteria were included in the study. According to the Rotterdam Criteria, PCOS is defined as the presence of two out of three characteristics i.e. oligo/anovulation, Clinical or Biochemical hyperandrogenism and polycystic ovarian morphology on Ultrasound [21]. The exclusion criteria was: Pregnancy, thyroid dysfunction, Hormone replacement therapy (HRT), malignancies, or any gynaecological condition other than PCOS.

### Outcome measurement

#### MPCOSQ

MPCOSQ is an updated and modified form of the PCOSQ. It consists of thirty items and seven subscales to assess the quality of life specific to PCOS. The subscales include emotional disturbances (7 items), hirsutism (5 items), infertility (3 Items), weight (6 Items), menstrual problems (3 Items), menstrual predictability (2 item) and acne (4 Items). Relevant items are summed to get subscale scores. All items are measured on a 7-point Likert scale, where “1” indicates maximum impairment and “7” indicates least impairment. MPCSOQ is a validated and reliable measure for the health-related quality of life of women with PCOS. [6, 16]

#### SF-36

**The** SF-36 was used to determine construct validity. The short form for HRQOL, the SF-36, is a commonly used measure designed for use across a wide range of conditions. It consists of eight subscales, including physical functioning (10 Items), role limitations due to physical issues (4 Items), body pain (2 Items), general perception of health (5 Items), social functioning (2 Items), role limitation due to emotional health (3 Items), vitality and mental health (5 Items). The scale is scored on a scale of 0-100, where higher scores indicate better quality of life. [22]

### Translation and cross-cultural adaptation

The MPCOSQ was translated and culturally adapted in Urdu language according to the guidelines of the World Health Organization (WHO) for tool translation. [23]

#### Step 1- Forward translation

Two forward translators, one of whom was a linguistic translator (Urdu scholar) and the second of whom was a bilingual medical expert who had command in both Urdu and English, translated the MPCOSQ into Urdu. The two Urdu versions (T1 & T2) were then reviewed by the primary investigator and the translators for meaning, relevance, structure and cultural sensitivity and combined into one prefinal Urdu version (T3).

#### Step 2- Backward translation

For backwards translation, a bilingual expert, blinded to the original MPCOSQ, was recruited to translate the final Urdu version (T3) of the MPCOSQ back to English. The backwards translation (BT3) along with the original version of the questionnaire were again reviewed by the same experts including the principal investigator. All the versions were reviewed for instructions’ similarity, format of items and responses, Sentence structure, relevance and meaning and incorporated into the prefinal version (T3).

#### Step 3- Pre testing and interviewing

This Prefinal Urdu version (T3) was then tested for face and content validity by ten experts who were gynaecologists with more than ten years of clinical experience. The expert panel suggested changes in the two translated versions of the MPCOSQ-U (T1 and T2), and the final version was developed after the revision based on the suggestions of the expert panel (T3). Revisions were suggested for Items 7, 19, 20 and 27 due to cultural differences. For item 22, the term “unsexy” was replaced with “not confident” due to the cultural nuances. Changes suggested by the expert panel were discussed with the Urdu scholar, and hence, the prefinal version (T3) of the MPCOSQ of Urdu was finalized.

A pilot testing was also done on a sample of 30 PCOS females for face validity asking about instructions, items, and clarity of responses. A verbal probing method was also used for the cognitive interviewing in which each respondent was asked whether the interviewer needed to repeat an item, difficulty in choosing the items or any clarification needed against each item. No amendments were suggested in the process. In the pretesting and cognitive interviewing, the participants reported that the questionnaire was easily understandable and included all the relevant items associated with PCOS. Therefore, no changes were made in the MPCOSQ-U after pretesting.

The prefinal version was then applied to a sample of 180 females with PCOS for psychometric analysis. Informed consent was taken prior to data collection.

### Data analysis procedure

The statistical analysis was performed using SPSS version 21.

### Validity

#### Content and face Validity

Content validity is defined as “the degree to which items in an instrument reflect the content.

universe to which the instrument will be generalized”. The ten Gynaecologists were asked to mark each item on a four-point Likert scale where “1” meant not relevant and “4” meant very relevant. Content Validity Ratio (CVR) and Content validity index (CVI) were then calculated using the Lawshe’s method. The minimum value of CVR to retain an item was 0.62 for 10 experts. [24] The acceptable limit for the CVI was set at 0.8. [25]

For face validity, a dichotomous scale of Yes and No was used and the expert panel of ten gynaecologists answered to this dichotomous scale against each item for terminology, adequacy, grammar, appropriateness and structure of items. [26]

### Construct validity

#### Factor analysis

Factor analysis determines the subscale factor structure of a tool. It simplifies the factor interpretation and reduces the number of items affected by each factor. The factor structure of MPCOSQ-U was explored using the principal component analysis (PCA) with varimax rotation keeping an eigenvalue of 1. Kaiser–Mayer–Olkin (KMO) measured the adequacy of sampling and a value > 0.6 indicated an adequate sample. For the Barlett’s test a p value of > 0.05 were considered significant [25]. A factor loading of ≥ 0.4 for the items was considered adequate. [27]

#### Concurrent validity

Concurrent validity was measured using Pearson’s correlation between the MPCOSQ-U and SF-36. A p value of 0.05 was considered significant. Portnoy and Watkins criteria were used to interpret the correlation, where *r* < .25 indicates little correlation, *r* = .25 to.5 indicates fair correlation, *r* = .5 to.75 indicates moderate correlation and *r* = .75 to 1 indicates good correlation. [28]

### Reliability analysis

#### Internal consistency and test-retest reliability

Reliability is defined as,” the extent to which the measurement of a variable is free from measurement error”. The reliability of MPCOSQ-U was measured using internal consistency and test-retest reliability. The internal consistency of MPCOSQ-U was assessed by calculating Cronbach’s alpha and taking responses on MPCOSQ-U by 180 respondents. A Cronbach’s alpha value of ≥ 0.7 was considered an acceptable internal consistency. [27]

For test-retest reliability, Intraclass correlation coefficient (ICC) was measured. Two measurements were taken on the MPCOSQ-U from thirty participants, which were two weeks apart for the test-retest reliability. The criteria used for the interpretation of ICC was categorized as: 0-0.2 small, 0.21 to 0.4 fair, 0.41–0.6 moderate, 0.61–0.8 substantial and 0.81 -1 high. [25]

## Results

### Psychometric analysis

#### Face and content validity

The responses of ten gynaecologists with at least 10 years of clinical experience were collected for face and content validity. The content validity ratio (CVR) of every item was above 0.8, and the content validity index (CVI) for the prefinal version was 0.92. Face Validity was assessed on a dichotomous scale of “Yes” and “No”. For all the items of the MPCOSQ-U, all experts responded with “Yes” to questions on terminology, adequacy, grammar, appropriateness, and structure of items.

### Test-retest reliability and internal consistency

**Fig. 1:**
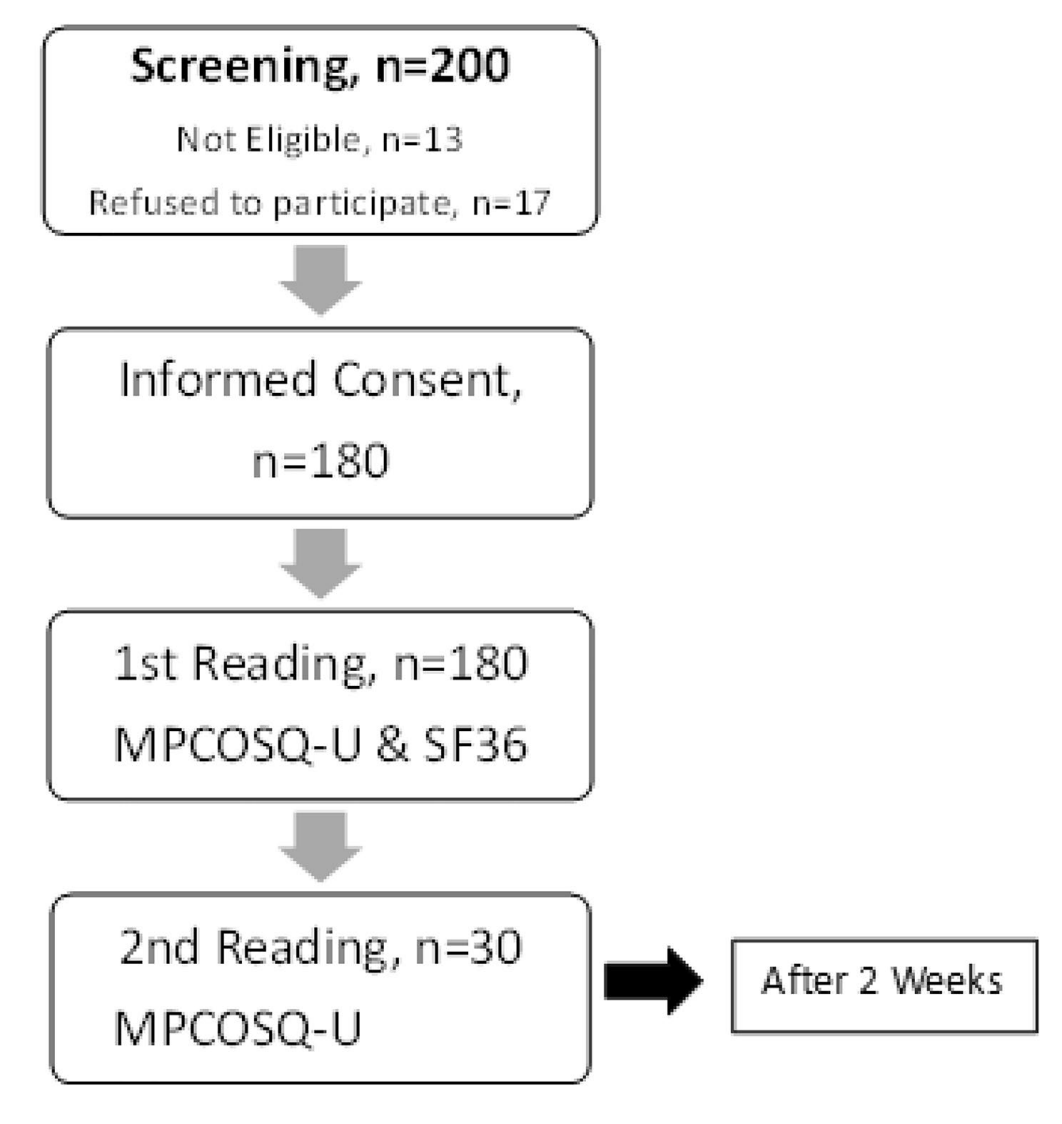
Flow chart of patient recruitment

Two hundred females with polycystic ovarian syndrome (PCOS) were screened on the eligibility criteria. Thirteen patients did not fulfil the eligibility criteria, and seven patients refused to participate. (Figure 1)

The average subscale scores and total scores as well as the reliability analysis are shown in Tables 1 and 2. The Urdu version of the MPCOSQ showed good internal consistency for all items (Cronbach’s alpha = 0.859). All subscales showed high internal consistency (Cronbach’s alpha = 0.795 to.850). A fair to good correlation was reported between individual items and total scores of the MPCOSQ-U with Spearman’s correlation coefficients of 0.396 to 0.852, which confirms the internal consistency of the MPCOSQ-U.

**Table 1:** Internal consistency reliability of the MPCOSQ-U

	Internal Consistency (*n* = 180)	
	Cronbach’s α	Mean(SD)
Emotions	0.795	32.21(9.66)
Hair	0.850	22.86(7.26)
Infertility	0.818	11.76(6.30)
Acne	0.823	18.90(5.70)
Weight	0.817	26.63(10.01)
Menstrual problems	0.809	24.52(6.30)
MPCOSQ-U Total Score	0.845	136.92(34.35)

Test-retest reliability was assessed by taking a second measurement after two weeks of the first from 30 respondents out of 180. The MPCOSQ-U showed excellent test-retest reliability for the total score (ICC_2, 1_=0.94) and subscales (ICC_2, 1_=0.93–0.99) (Table 2).

**Table 2:** Test-retest reliability of the MPCOSQ-U

MPCOSQ-U	Test retest reliability (*n* = 30)		
	1st Measurement Mean(SD)	2nd Measurement Mean(SD)	ICC (95% CI)
Emotions	27.86(10.84)	27.96(10.43)	0.993*
Hair	20.40(8.84)	21.03(8.02)	0.976*
Infertility	8.76(5.44)	9.40(5.16)	0.982*
Acne	18.83(6.97)	19.36(7.71)	0.987*
Weight	22.53(10.11)	19.37(7.71)	0.983*
Menstrual problems	23.90(7.21)	23.70(6.96)	0.991*
Total Score	122.30(39.41)	120.26(35.81)	0.939*

* *p* < .05; SD: standard deviation, CI: confidence interval.

### Factor analysis

The results of a Kaiser–Meyer–Olkin (KMO) measure of sampling adequacy showed a satisfactorily high (0.907) KMO value. Bartlett’s test was significant (*p* ≤ .05), which indicates that all items were correlated. A six-factor structure was demonstrated based on an eigenvalue of > 1. The PCA revealed six factors for the MPCOSQ-U. The loading factor for all items was greater than 0.4 (Table 3).

**Table 3:** Principal component analysis of MPCOSQ-U

Items		Subscales					
		Emotional Disturbance	Acne	Weight	Infertility	Hirsuitism	Menstrual Problems
Q2	Feel depressed as a result of having PCOS	0.846					
Q17	Feel worried about having PCOS	0.836					
Q18	Feel self-conscious as a result of having PCOS	0.810					
Q6	Feel moody as a result of having PCOS	0.755					
Q11	Experienced low self-esteem as a result of having PCOS	0.699					
Q23	Feel a lack of control over the situation with PCOS	0.669					
Q24	Have difficulties staying at your ideal weight		0.858				
Q10	Had trouble dealing with your weight		0.847				
Q12	Feel frustration in trying to lose weight		0.819				
Q3	Feel concerned about being overweight		0.760				
Q22	Feel like you are not confident because of being overweight		0.760				
Q4	Tired easily		0.437				
Q15	Growth of visible hair on your face			0.842			
Q26	Growth of visible body hair			0.814			
Q9	Growth of visible hair on your upper lip			0.810			
Q1	Growth of visible hair on your chin			0.793			
Q16	Feelings of embarrassment about excessive body hair			0.766			
Q8	irregular menstrual bleeding				0.740		
Q7	Headaches				0.732		
Q20	late menstrual period				0.696		
Q19	abdominal bloating				0.599		
Q21	menstrual cramps				0.494		
Q25	Feel sad because of infertility problems					0.779	
Q13	Feel afraid of not being able to have children					0.767	
Q5	Feel concerned about infertility problems					0.754	
Q14	Feel afraid of getting cancer					0.578	
Q29	Feel depressed as a result of acne						0.746
Q28	Feel unattractive because of acne						0.671
Q27	Acne (last 2 weeks)						0.600
Q30	Acne in last menstrual period						0.575

**Fig. 2:**
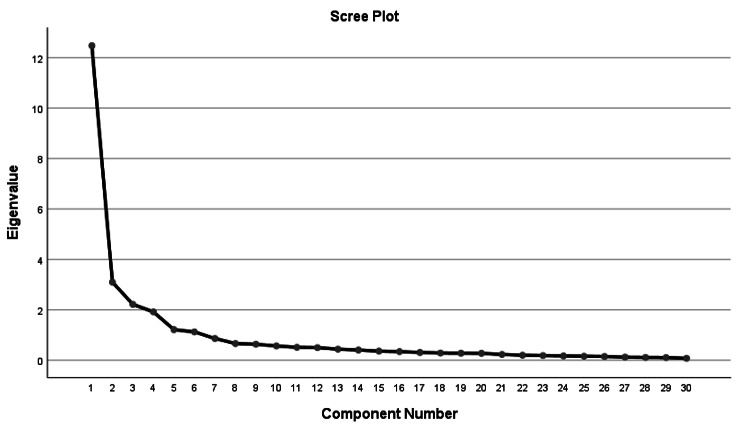
Scree plot showing the six-factor structure of the MPCOSQ-U

The eigenvalue for the first factor was 41.01, which explained 42.38% of the variance. The total variance explained by all six factors was 74.28% (Table 4). The six-factor structure is supported by a scree plot that depicts the straightening of the line after the first six factors. (Figure 2)

**Table 4:** Total Variance explained

MPCOSQ-U	% of variance
Emotional disturbance	42.38%
Hirsuitism	10.58%
Infertility	7.11%
Acne	5.84%
Weight	4.73%
Menstrual problems	3.64%
Total	74.28%

### Concurrent validity

Pearson’s correlation showed a statistically significant but weak positive correlation between the total scores of the MPCOSQ-U and SF-36 (*n* = 180, *r* = .186, *p* = .012).

## Discussion

The MPCOSQ has been translated into multiple languages, but this is the first study that has translated the MPCOSQ to the Urdu. The MPCOSQ-U is a valid and reliable tool to determine the quality of life of females with polycystic ovary syndrome. All items of the MPCOSQ-U were culturally relevant to Urdu-speaking Pakistani females with PCOS. The current study showed that the MPCOSQ-U is easy to understand and can be used in clinical settings in Pakistan.

The version used for translation had a seven-factor structure which is also reported by other translated versions. For the seven-domain structure the domain of “Menstrual factors” is split into “menstrual symptoms” and “menstrual predictability”. [29–31]

A six-factor structure was identified for the Urdu version of MPCOSQ with a slight difference in the item loading for the emotional and infertility domains. The item “Feel afraid of getting cancer” loaded on the infertility subscale, which was previously in the domain of emotional disturbances. Previously a seven factor structure has been reported by the original MPCOSQ and chi-MPCOSQ [17], however the Iranian version reported a six factor structure. [6]

The MPCOSQ has also been translated into Marathi language. It showed a high content validity, and the Cronbach’s alpha for this version was 0.92 showing good reliability. [32]

The results of the current study are comparable to the previous literature. The Arabic version of the MPCOSQ reported a CVI of 0.9 from ten experts, which shows acceptable content validity. Cronbach’s alpha coefficients demonstrated good internal consistency for all items together (α = 0.863). The ICC for each subscale ranged from 0.911 to 0.986 [25]. The current study also reported a high internal consistency (CVI = 0.91) and test-retest reliability (ICC = 0.939).

A Chinese version of the MPCOSQ showed 77% overall variance and had an internal consistency of 0.88 and a good test-retest reliability of 0.89, which overall demonstrates its good discriminant validity. There was an addition of another subscale of “Menstrual predictability” to the Chinese version of the MPCOSQ and the Chi-MPCOSQ had a seven-factor structure. This is in contrast to our results, where MPCOSQ-U showed a six factor structure. [30]

The MPCOSQ has also been translated into Dutch. The Dutch version of the tool demonstrated excellent internal consistency (α = 0.95) and a high test-retest reliability for all subscales of the MPCOSQ (ICC: 0.88–0.96). The factor analysis, however, did not confirm the six-factor structure, and another domain was added for “coping”. Overall, it was reported to be a reliable and disease-specific measure for women with PCOS. It was recommended that the item “lack of control over situation with PCOS” be moved to the emotional domain. [29] However, contrary to this finding, the current study showed the same item loading on the emotional subscale.

Bazarganipour et al. translated the MPCOSQ to the Iranian language. The psychometric analysis was performed on a sample of 200 Iranian females. The Iranian version of the MPCOSQ showed excellent content validity (CVI: 0.96). The scale also showed a six-factor structure on exploratory factor analysis. The item “late menstrual period” was loaded for the menstrual subscale instead of emotional disturbance. The CFA also supported that the six-factor structure was a fit to the model. The internal consistency and intraclass correlation coefficient (ICC) were also satisfactory. All subscales were significantly correlated. [6]

The main strength of this study is that it fills an important gap of addressing the requirement of culturally appropriate assessment tool for PCOS, and therefore making the tool accessible to Urdu-speaking population. Since PCOS is on the rise in Pakistan, this Urdu translated version will be beneficial in assessing the PCOS specific quality of life and thereby help females improve the symptoms and assess the impact of interventions.

The main limitation of the study is that the SF-36 was used as a comparison tool for concurrent validity. Although this tool is a widely used measure of quality of life related to health, it may still have limitations in capturing the specific aspects of PCOS that measured by the MPCOSQ-U. Using a more disease-specific comparison tool is recommended as it could provide a more robust assessment of construct validity. A weak correlation between MPCOSQ-U and SF-36 (*r* = .186), which could raise concerns regarding the concurrent validity of the MPCOSQ-U. Further investigations are needed to explore the psychometric properties of MPCOSQ-U by including a diverse group of experts and patients to make sure that it accurately reflects the experiences of Pakistani women with PCOS.

## Conclusion

The MPCOSQ-U is a validated and reliable tool to assess the quality of life of Urdu-speaking Pakistani females with PCOS. This is an important step to cover the language barrier, which influences the outcome assessment in PCOS. Since Pakistani population is diverse, with a majority with an understanding of Urdu Language, cross-cultural comparative studies should be conducted across various cultural groups of Pakistan which would require the translation of MPCOSQ to other languages used in Pakistan.

## Data Availability

Manuscript does not contain any material from third party. All the material is owned by the authors and no permissions are required.
